# Associations of chronic obstructive pulmonary disease with life satisfaction and self-rated health: quantifying the subjective burden in a population-based study

**DOI:** 10.1007/s11136-025-04112-9

**Published:** 2025-12-23

**Authors:** Yusuff Adebayo Adebisi, Najim Z. Alshahrani, Godino Kalungi, Okoye Stephanie Somtochukwu, Don Eliseo III Lucero-Prisno

**Affiliations:** 1 College of Social Sciences, University of Glasgow, 40 Bute Gardens, Glasgow, G12 8RT UK; 2 Department of Family and Community Medicine, Faculty of Medicine, University of Jeddah, Jeddah, Saudi Arabia; 3 Urology Resident, Frimley Park Hospital, NHS, Frimley, UK; 4 Franklin University, Columbus, OH USA; 5 Federal Medical Centre, Ebute Metta, Lagos, Nigeria; 6 Department of Global Health and Development, London School of Hygiene and Tropical Medicine, London, UK

**Keywords:** COPD, Life satisfaction, Self-rated health, Subjective wellbeing, Population health, Deprivation, Scotland, Scottish Health Survey

## Abstract

**Introduction:**

Subjective health and wellbeing measures such as self-rated health and life satisfaction are widely used in population health research and surveillance, yet their ability to reflect the lived burden of chronic disease has rarely been tested directly. This cross-sectional study examined whether life satisfaction and self-rated health differ between individuals with and without chronic obstructive pulmonary disease (COPD).

**Methods:**

We used pooled data from the 2018, 2019, 2021, and 2022 waves of the Scottish Health Survey, comprising 18,563 adults aged 16 years and over. COPD status was determined through self-report of a doctor diagnosis. Life satisfaction was categorised as low (0–7), moderate (8), or high (9–10), and self-rated health was grouped as very good/good, fair, or bad/very bad. Ordinal logistic regression was used to estimate unadjusted associations and generalised ordered logistic regression was applied for adjusted models, controlling for age, sex, smoking, ethnicity, education, mental health, area deprivation, and urban/rural residence.

**Results:**

Individuals with COPD had significantly poorer subjective wellbeing. In unadjusted models, individuals with COPD had 60% lower odds of reporting higher life satisfaction (OR = 0.40; 95% CI 0.35–0.46) and nearly nine times the odds of reporting worse self-rated health (OR = 8.70; 95% CI 7.64–9.92) compared to those without COPD. After adjustment, these associations remained strong: COPD was associated with 49% lower odds of higher life satisfaction (OR = 0.51; 95% CI 0.44–0.59) and over fourfold higher odds of poor self-rated health (OR = 4.24; 95% CI 3.68–4.89).

**Conclusions:**

Self-rated health and life satisfaction clearly distinguish individuals with COPD from those without, supporting their construct validity as indicators of chronic disease burden in population-based surveys. These brief measures may offer valuable tools for public health monitoring and evaluation of health inequalities.

## Introduction

Subjective wellbeing has become an increasingly important focus in public health, reflecting recognition that health encompasses physical, psychological, and social dimensions [1–3]. It is now widely accepted that health cannot be fully understood through clinical indicators alone [4–6]. Instead, how individuals perceive and evaluate their own health and quality of life is essential for informing policy and service delivery [7]. In line with recommendations from international organisations such as the World Health Organization [8] and the Organisation for Economic Co-operation and Development [9], population surveys increasingly include brief self-report measures of subjective wellbeing and perceived health status—most commonly life satisfaction as an indicator of overall wellbeing and self-rated health as a global assessment of personal health. Both measures are routinely included in population health surveys and have demonstrated utility for predicting mortality, healthcare utilisation, and future health decline [8].

Despite their widespread adoption, questions remain about the validity of these single-item measures in accurately capturing the lived experience of chronic illness. While self-rated health and life satisfaction are often assumed to reflect underlying wellbeing and health status [9–13], this assumption has rarely been tested directly using well-defined chronic disease groups. Chronic obstructive pulmonary disease (COPD) offers an appropriate context to examine this issue. COPD is a progressive respiratory condition that leads to persistent symptoms such as breathlessness, cough, and fatigue and often results in functional impairment, psychological distress, social isolation, and diminished quality of life [14]. Given these impacts, individuals with COPD would be expected to report substantially lower levels of subjective wellbeing and health status. Empirical confirmation of this pattern would strengthen confidence in the use of self-rated health and life satisfaction as indicators of health burden in population-level surveillance and research.

Although numerous studies have documented the negative impact of COPD on physical functioning and health-related quality of life, most have relied on disease-specific instruments such as the St George’s Respiratory Questionnaire or the COPD Assessment Test [14]. Far fewer have examined whether general population indicators, such as self-rated health and life satisfaction, accurately mirror this burden. Establishing such correspondence is important for validating the use of brief wellbeing and health indicators in national surveys, particularly when condition-specific assessments are unavailable. Evaluating the sensitivity of subjective wellbeing measures to a condition such as COPD is particularly relevant for informing health inequality monitoring. COPD disproportionately affects older adults, people from socioeconomically disadvantaged backgrounds, and those with a history of smoking. If subjective wellbeing and health indicators consistently reflect poorer outcomes in these populations, they can serve as valuable tools for assessing the distribution of health burden and targeting interventions [8, 9]. Moreover, since these indicators are brief and cost-effective to collect, they offer practical advantages for repeated measurement in national surveys.

This study used data from four recent waves of the Scottish Health Survey to examine whether two commonly used subjective measures, self-rated health and life satisfaction, are associated with the presence of COPD, thereby assessing their construct validity as indicators of subjective health burden. We assessed whether individuals with COPD report significantly poorer outcomes on these indicators, even after accounting for sociodemographic characteristics, smoking status, and mental health. We also examined whether these associations differ across levels of area deprivation.

## Methods

### Data source and sample selection

This cross-sectional analysis used data from the Scottish Health Survey (SHeS), an annual, nationally representative survey commissioned by the Scottish Government to monitor the health and health-related behaviours of the Scottish population [15]. The survey uses a stratified, multistage probability sampling design to produce representative estimates at both national and regional levels [16]. Data are collected through face-to-face interviews and self-completion questionnaires, covering a wide range of topics including general health, lifestyle behaviours, diagnosed conditions, and subjective wellbeing [15]. The SHeS is widely used in public health research and is a key component of Scotland’s national health monitoring framework [15, 16]. As a repeated cross-sectional survey, each annual wave comprises a new, independent sample of adults rather than follow-ups of the same individuals. Respondents are not tracked across years, and no unique identifiers are used to link participants between survey waves.

We pooled data from four recent waves of the survey: 2018, 2019, 2021, and 2022. The 2020 wave was excluded due to limited data collection during the COVID-19 pandemic and lack of comparability with other years [16]. The 2021 survey was conducted primarily by telephone, whereas the 2022 wave returned to in-home, face-to-face interviewing using standard fieldwork protocols and weighting procedures designed to restore national representativeness. According to the Scottish Government’s official reports, both waves applied weighting adjustments to maintain comparability with pre-pandemic years, although caution is warranted when interpreting data from 2021 due to its alternative collection mode [17]. Pooling multiple years allowed for a larger sample size and increased precision in subgroup analyses.

The combined dataset initially included 25,986 individuals across the four waves. We excluded respondents not eligible for the adult health modules (e.g. children or those flagged as ‘Schedule not applicable’; *n* = 7322). Participants with missing or non-informative responses on key variables were then removed: sex (‘Prefer not to say’ or ‘Refused’; *n* = 41), life satisfaction (‘Refused’ or ‘Don’t know’; *n* = 55), self-rated health (‘Don’t know’; *n* = 4), and COPD diagnosis (‘Don’t know’; *n* = 1). The final analytic sample comprised 18,563 adults aged 16 years and older with complete data on COPD status, life satisfaction, and self-rated health. Missing or non-informative responses represented < 1% of the pooled dataset, supporting the use of complete-case analysis.

## Exposure and outcome measures

The primary exposure variable in this study was COPD, ascertained through self-report. Respondents were asked whether they had ever been told by a doctor that they had COPD. This approach reflects clinical diagnosis as understood and recalled by the participant. Responses were coded as “COPD” or “No COPD.” Participants who responded “don’t know” were excluded from the analysis due to lack of diagnostic certainty.

Two outcomes were analysed as indicators of subjective wellbeing and health: life satisfaction and self-rated health.

Life satisfaction was assessed using a single-item question: “All things considered, how satisfied are you with your life as a whole nowadays?” Respondents rated their satisfaction on a scale from 0 to 10, where 0 indicated “extremely dissatisfied” and 10 indicated “extremely satisfied.” For the main analysis, scores were categorised into three ordered groups based on their position relative to the mode of the distribution: low satisfaction (scores 0–7), moderate satisfaction (score of 8, which was the modal value), and high satisfaction (scores 9–10). This categorisation is consistent with the approach used in the Scottish Health Survey annual reports, which apply the same grouping to reflect the skewed distribution of responses and the strong clustering at a score of 8.

Self-rated (subjective) health was measured using the question, “How is your health in general?” with five response options: very good, good, fair, bad, and very bad. These responses were grouped into three ordinal categories: very good/good (coded as 1), fair (coded as 2), and bad/very bad (coded as 3), with higher values indicating poorer health.

## Covariates

Covariates were selected a priori based on theoretical relevance and empirical evidence linking them to both COPD and subjective health and wellbeing [18, 19]. These covariates accounted for key sociodemographic, behavioural, and health-related factors that could confound the association between COPD and the two outcome measures. Age was categorised into four groups: 16–34, 35–54, 55–64, and 65 years and older. Sex was coded as male or female. Smoking status was classified into four categories based on self-reported smoking history: never smoker, ex-smoker (occasional), ex-smoker (regular), and current smoker. Ethnicity was dichotomised as White or Non-White due to small numbers in minority ethnic groups. Educational attainment was grouped into three categories: higher education (e.g. university degree or equivalent), secondary or college level qualification, and lower or no qualification. Area-level deprivation was measured using the Scottish Index of Multiple Deprivation 2020 (SIMD). Urban or rural residence was included based on the respondent’s postcode classification, categorised as urban or rural. Finally, the presence of a self-reported mental health condition was included as a binary variable (yes or no), based on whether respondents reported being diagnosed with depression, anxiety, or another mental health condition.

### Statistical analysis

We began by describing sample characteristics stratified by COPD status, using frequencies and percentages for categorical variables. Group differences between individuals with and without COPD were assessed using Pearson’s chi-square tests of independence.

To examine the association between COPD and each subjective outcome, we treated both life satisfaction and self-rated health as ordinal dependent variables and applied appropriate regression models. For the crude analyses, ordinal logistic regression (ologit) was used to estimate unadjusted odds ratios (ORs) with 95% confidence intervals (CIs). The proportional-odds assumption was formally tested and not violated for either outcome, supporting the use of standard ordinal logistic models for the unadjusted analyses. For adjusted analyses, we applied generalised ordinal logistic regression models, implemented in Stata using the gologit2 command with the autofit option. This model relaxes the proportional-odds assumption for covariates that violate it, while retaining it for others, allowing more accurate estimation when the parallel-lines assumption does not hold uniformly across predictors [20].

All adjusted models controlled for relevant sociodemographic, behavioural, and health-related covariates identified a priori. These included age group, sex, smoking status, ethnicity, education, area-level deprivation, urban or rural residence, and the presence of a self-reported mental health condition. For life satisfaction, higher scores reflect better wellbeing; therefore, an OR < 1 for COPD indicates lower odds of reporting higher life satisfaction. In contrast, for self-rated health, higher scores represent poorer health; thus, an OR > 1 for COPD indicates higher odds of reporting worse health. To retain the full analytic sample and minimise bias due to missing data, participants with missing values for categorical covariates (smoking status, ethnicity, or education) were included in the models as separate ‘missing’ categories.

As a sensitivity analysis, we adjusted for survey year (2018, 2019, 2021, and 2022) to account for potential period effects and differences in data-collection mode across survey waves.

To further explore the intersection of COPD and area-level deprivation, we generated predicted probabilities for subjective wellbeing and subjective health status using the margins command in Stata, stratified by COPD status and deprivation quintile. These estimates were derived from the fully adjusted generalised ordered logistic regression models. A likelihood-ratio test (LRT) was also performed to formally assess the presence of interaction between COPD and deprivation quintile. All analyses were conducted in Stata version 18 (StataCorp, College Station, TX), with statistical significance assessed at the 5% level.

## Results

Table 1 presents the sociodemographic and health-related characteristics of the study population (*n* = 18,563), stratified by COPD status. Among the 855 individuals with COPD, a markedly older age profile was observed compared to those without the condition: 61.5% were aged 65 and above, compared to 30.0% in the non-COPD group (*p* < 0.001). Sex distribution was similar between groups (*p* = 0.535), with women comprising 57.2% of the COPD group and 56.1% of those without COPD. Marked differences emerged in smoking status (*p* < 0.001). Nearly half of individuals with COPD were regular ex-smokers (46.1%), and over a third were current smokers (35.9%), compared to 24.2% and 13.2%, respectively, in those without COPD. Individuals with COPD were also significantly less likely to have never smoked (16.3% vs. 55.7%). Educational attainment and area deprivation differed significantly by COPD status (both *p* < 0.001). More than half (52.1%) of those with COPD had lower or no educational qualifications, compared to 17.1% among those without COPD. Similarly, 32.2% of individuals with COPD lived in the most deprived SIMD quintile, while only 11.1% were in the least deprived category. Mental health conditions were more prevalent among those with COPD (18.6%) compared to those without (10.6%; *p* < 0.001). Urban residence was slightly more common in the COPD group (84.0%) than in the non-COPD group (77.9%; *p* < 0.001).

**Table 1:** Characteristics of the sample by COPD status (*n* = 18,563)

Characteristic	No COPD (*n* = 17,708)	COPD (*n* = 855)	Total (*n* = 18,563)	*p*-value
*Age group*,* n (%)*				< 0.001
16–34	3472 (19.6)	13 (1.5)	3485 (18.8)	
35–54	5572 (31.5)	104 (12.2)	5676 (30.6)	
55–64	3361 (19.0)	212 (24.8)	3573 (19.2)	
65+	5303 (30.0)	526 (61.5)	5829 (31.4)	
*Sex*,* n (%)*				0.535
Male	7771 (43.9)	366 (42.8)	8,137 (43.8)	
Female	9937 (56.1)	489 (57.2)	10,426 (56.2)	
*Smoking status*,* n (%)*				< 0.001
Never smoked	9867 (55.7)	139 (16.3)	10,006 (53.9)	
Ex-smoker (occasional)	1100 (6.2)	15 (1.8)	1115 (6.0)	
Ex-smoker (regular)	4282 (24.2)	394 (46.1)	4,676 (25.2)	
Current smoker	2341 (13.2)	307 (35.9)	2,648 (14.3)	
Missing	118 (0.7)	0 (0.0)	118 (0.6)	
*Ethnicity*,* n (%)*				< 0.001
White	16,918 (95.5)	852 (99.6)	17,770 (95.7)	
Non-White	746 (4.2)	2 (0.2)	748 (4.0)	
Missing	44 (0.2)	1 (0.1)	45 (0.2)	
*Education*,* n (%)*				< 0.001
Higher education	9199 (52.0)	211 (24.7)	9,410 (50.7)	
Secondary/College qualification	5408 (30.5)	197 (23.0)	5605 (30.2)	
Lower/No qualification	3033 (17.1)	445 (52.1)	3478 (18.7)	
Missing	68 (0.4)	2 (0.2)	70 (0.4)	
*Deprivation*,* n (%)*				< 0.001
Least deprived	3814 (21.5)	95 (11.1)	3909 (21.1)	
4th quintile	4122 (23.3)	132 (15.4)	4254 (22.9)	
3rd quintile	3767 (21.3)	148 (17.3)	3915 (21.1)	
2nd quintile	3347 (18.9)	205 (24.0)	3552 (19.1)	
Most deprived	2658 (15.0)	275 (32.2)	2933 (15.8)	
*Urban/Rural*,* n (%)*				< 0.001
Urban	13,787 (77.9)	718 (84.0)	14,505 (78.1)	
Rural	3,921 (22.1)	137 (16.0)	4058 (21.9)	
*Mental health condition*,* n (%)*				< 0.001
No condition	15,840 (89.5)	696 (81.4)	16,536 (89.1)	
Has condition	1868 (10.6)	159 (18.6)	2027 (10.9)	
*Self-rated health*,* n (%)*				< 0.001
Very good/Good	12,873 (72.7)	196 (22.9)	13,069 (70.4)	
Fair	3457 (19.5)	301 (35.2)	3758 (20.2)	
Bad/Very bad	1378 (7.8)	358 (41.9)	1736 (9.4)	
*Life satisfaction*,* n (%)*				< 0.001
Low (0–7, below mode)	5957 (33.6)	494 (57.8)	6451 (34.8)	
Moderate (8, mode)	5366 (30.3)	178 (20.8)	5544 (29.9)	
High (9–10, above mode)	6385 (36.1)	183 (21.4)	6568 (35.4)	

Percentages are column percentages. *p*-values from Pearson Chi-square tests of independence

Table 2 presents the crude and adjusted odds ratios for the associations between COPD and the two subjective indicators, life satisfaction and self-rated health, among adults in Scotland. In the unadjusted model, individuals with COPD had 60% lower odds of reporting higher life satisfaction compared with those without COPD (crude OR = 0.40; 95% CI 0.35–0.46; *p* < 0.001). After adjustment for age, sex, smoking status, ethnicity, education, area-level deprivation, urban/rural residence, and mental health condition, the association remained strong and statistically significant (adjusted OR = 0.51; 95% CI 0.44–0.59; *p* < 0.001). For self-rated health, individuals with COPD had substantially higher odds of reporting poorer health in the crude analysis (OR = 8.70; 95% CI 7.64–9.92; *p* < 0.001). After controlling for the same covariates, the association remained robust (adjusted OR = 4.24; 95% CI 3.68–4.89; *p* < 0.001). The results presented are from models without survey-year adjustment; however, a sensitivity analysis that included survey year (2018–2022) yielded similar estimates, with no material change in the direction or magnitude of the associations.

**Table 2:** Crude and adjusted odds ratios for the association between COPD and subjective outcomes (*n* = 18,563)

Outcome (Ordinal scale)	Model type	Odds ratio (95% CI)	*p*-value
Life satisfaction (Low = 1, Moderate = 2, High = 3)	Crude model (ordinal logit)	0.40 (0.35–0.46)	< 0.001
	Adjusted model (Generalised ordered logit)¹	0.51 (0.44–0.59)	< 0.001
Self-rated health (1 = Very good/Good, 2 = Fair, 3 = Bad/Very bad)	Crude model (ordinal logit)	8.70 (7.64–9.92)	< 0.001
	Adjusted model (Generalised ordered logit)¹	4.24 (3.68–4.89)	< 0.001

¹Adjusted for age, sex, smoking status, ethnicity, education, area-level deprivation, urban/rural residence, and mental health condition.

OR < 1 indicates lower odds of reporting higher life satisfaction; OR > 1 indicates higher odds of poorer self-rated health. The proportional odds assumption held for COPD under autofit, so a single OR is reported.

**Fig. 1:**
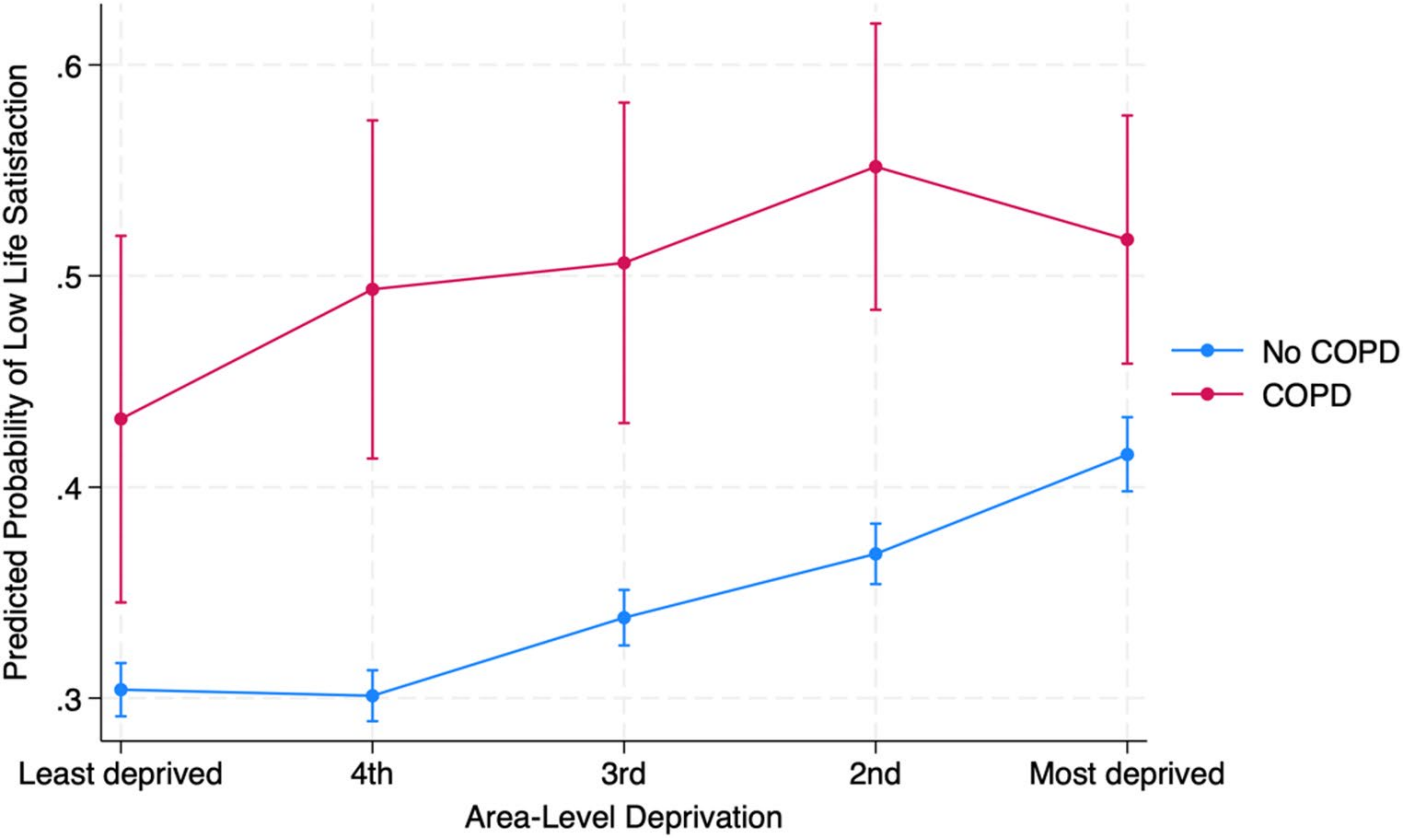
Predicted probability of low life satisfaction by COPD and SIMD Quintile. *Note* Low life satisfaction was defined as scores between 0 and 7 on a 0–10 scale (higher values indicate greater satisfaction). Predicted probabilities were estimated from fully adjusted generalised ordinal logistic regression models. Error bars represent 95% confidence intervals

Figure 1 shows the predicted probability of reporting low life satisfaction (score 0–7) across area-level deprivation quintiles, stratified by COPD status. In all deprivation groups, individuals with COPD had a significantly higher probability of low life satisfaction compared to those without COPD (all *p* < 0.001). Among the least deprived, the probability of low life satisfaction was 43.2% (95% CI 34.5–51.9) for individuals with COPD, compared to 30.4% (95% CI 29.1–31.7) for those without—an absolute difference of 12.8% points. The disparity peaked in the 4th deprivation quintile, where 49.4% (95% CI 41.4–57.4) of individuals with COPD reported low satisfaction versus 30.1% (95% CI 28.9–31.3) of those without, yielding a 19.3% point difference. While the probability of low life satisfaction increased with deprivation in both groups, the gap between COPD and non-COPD respondents remained broadly similar, ranging from 10.2 to 19.3% points. A likelihood ratio test comparing models with and without the interaction term (COPD × SIMD) indicated no significant effect modification by deprivation (LR χ²(4) = 5.58, *p* = 0.23).

**Fig. 2:**
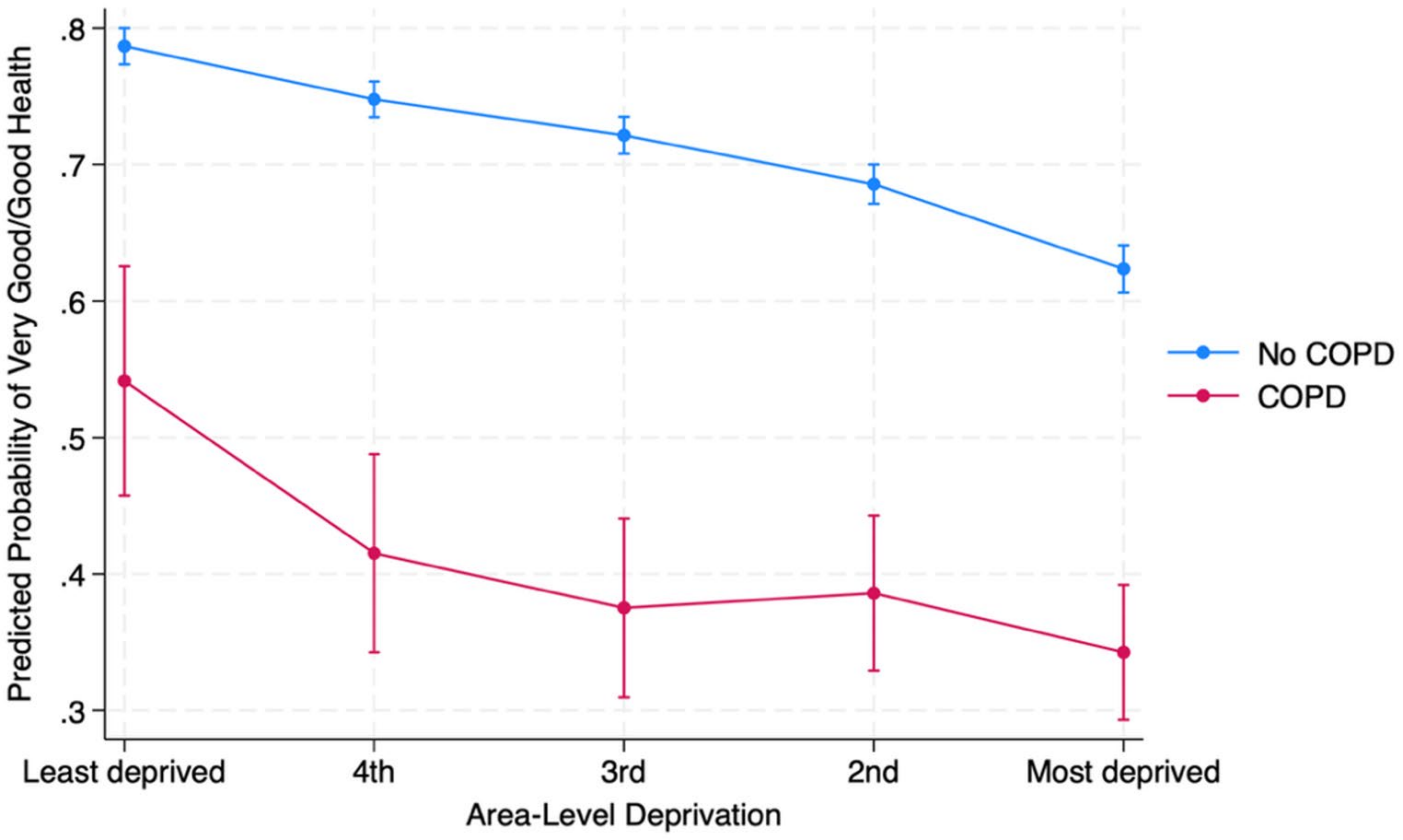
Predicted probability of very good/good health by COPD and SIMD Quintile. *Note* Very good/good self-rated health was defined as responses of “very good” or “good” on a five-point scale (very good, good, fair, bad, very bad). Predicted probabilities were derived from fully adjusted generalised ordinal logistic regression models. Error bars represent 95% confidence intervals

Figure 2 presents the predicted probability of reporting very good or good self-rated health across deprivation quintiles, stratified by COPD status. Across all deprivation levels, individuals with COPD were substantially less likely to report favourable health than those without the condition (all *p* < 0.001). In the least deprived group, 78.7% (95% CI 77.3–80.0) of non-COPD respondents reported good or very good health, compared to 54.1% (95% CI 45.7–62.5) of those with COPD—a 24.6% point gap. The disparity widened further in middle quintiles, peaking in the 3rd quintile with a 34.7% point gap (72.2% vs. 37.5%). Among the most deprived, 62.4% (95% CI 60.6–64.1) of non-COPD individuals reported very good or good health, while only 34.3% (95% CI 29.3–39.2) of individuals with COPD did so—a 28.1% point difference. To formally assess whether the relationship between COPD and self-rated health differed by deprivation level, an interaction term (COPD × SIMD) was included in the model. The interaction was not statistically significant (LR χ²(4) = 4.62, *p* = 0.33).

## Discussion

This study provides evidence that individuals living with COPD report significantly poorer subjective wellbeing and health status, as measured by both self-rated health and life satisfaction. These findings are consistent with the clinical understanding of COPD as a debilitating chronic condition marked by persistent breathlessness, fatigue, and limitations in daily functioning [21]. Importantly, even after adjusting for smoking status, sociodemographic factors, and mental health conditions, individuals with COPD had more than four times the odds of reporting poor self-rated health and roughly half the odds of reporting higher life satisfaction. Although the primary aim of this study was to examine these associations rather than to validate the measures themselves, the strength and consistency of the findings suggest that these brief, single-item indicators can meaningfully reflect the lived burden of chronic disease. Prior studies have shown that poor self-rated health predicts hospitalisation, functional decline, and mortality [22–25]; our results extend this evidence by demonstrating that these wellbeing measures can clearly distinguish between individuals with and without a chronic respiratory condition such as COPD. The consistent patterns across both wellbeing outcomes further strengthen confidence in their utility as concise, population-relevant public health indicators.

Furthermore, individuals with COPD had lower levels of wellbeing across all levels of deprivation, and predicted probabilities of poor outcomes were markedly higher in the most deprived groups. These patterns reflect the well-documented concentration of COPD in socioeconomically disadvantaged populations, driven by higher rates of smoking, occupational exposures, and limited access to timely diagnosis and care [26]. The consistently poorer wellbeing observed among individuals with COPD across the deprivation gradient suggests that COPD exerts a substantial, independent effect on subjective experience, regardless of material context. Although deprivation itself was strongly associated with reduced wellbeing, the lack of a significant interaction between COPD and deprivation indicates that the detrimental effects of COPD on wellbeing were broadly similar across socioeconomic strata. However, COPD remains disproportionately concentrated in deprived communities, meaning that population-level burdens are amplified by social disadvantage [27–29].

While the use of single-item measures such as self-rated health and life satisfaction offers clear advantages in terms of simplicity, comparability, and feasibility for large-scale population surveys, they inevitably capture only a subset of the broader dimensions of wellbeing. Multi-item instruments such as the EQ-5D and WHOQOL provide richer assessments of specific domains including physical function, social participation, and psychological wellbeing [30–32]. However, our findings indicate that even brief, single-item questions are sufficiently sensitive to distinguish between individuals with and without COPD, suggesting that they remain valuable tools for public health monitoring where brevity and respondent burden are key considerations [33]. Future research may build on these findings by comparing the performance of single- and multi-item measures within the same populations to assess their relative validity and responsiveness.

These findings have clear implications for the use of self-rated health and life satisfaction as monitoring tools in public health research. First, they offer empirical support for the construct validity of these single-item measures as indicators of disease burden, especially in chronic conditions like COPD where functional impairment and emotional distress are prominent. Researchers and policymakers can be more confident in using these indicators to track health trends, allocate resources, and evaluate the population-level impact of chronic diseases. Second, the study illustrates the utility of generalised ordered logit models for testing the proportional odds assumption in ordinal outcomes, a methodological approach that may be underused in health inequalities research. Finally, the robustness of associations after adjusting for confounders suggests that these measures may serve as equity-sensitive metrics, capable of capturing disparities in health experience that might be missed by clinical or administrative records. Future research could explore their performance in other disease groups, their responsiveness to intervention, and their predictive value for long-term outcomes.

A major strength of this study is the use of nationally representative data from four recent waves of the Scottish Health Survey, providing sufficient statistical power and generalisability to the adult population of Scotland. The inclusion of both self-rated health and life satisfaction offers a broader assessment of subjective wellbeing than studies relying on a single indicator. The use of generalised ordered logit models represents a methodological advance by accommodating partial violations of the proportional odds assumption, thereby yielding more accurate estimates.

However, several limitations should be acknowledged. The identification of COPD was based on self-report of a prior clinical diagnosis, which may be prone to recall error or underreporting, particularly among individuals with limited access to healthcare. Such misclassification is likely to be non-differential and would therefore bias the observed associations toward the null, implying that the true relationship between COPD and subjective wellbeing may be even stronger than reported. Given the cross-sectional design, causality cannot be inferred [34], and the temporal sequence between COPD and wellbeing remains unclear. It is also possible that poorer wellbeing could influence the reporting or perception of respiratory symptoms or increase healthcare-seeking behaviour, leading to higher likelihood of COPD diagnosis—introducing potential reverse causation. Moreover, the survey was not designed specifically for this analysis, limiting access to clinical indicators such as disease severity or lung function measures. While the models adjusted for key sociodemographic, behavioural, and mental health covariates, residual confounding by unmeasured factors cannot be ruled out. Despite these limitations, the consistency and magnitude of associations observed across wellbeing measures provide strong evidence that COPD is associated with substantially poorer perceived health and life satisfaction in the Scottish adult population.

## Conclusion

This study provides clear evidence that individuals living with chronic obstructive pulmonary disease experience significantly poorer subjective health and wellbeing, as reflected in both self-rated health and life satisfaction. These associations remained strong even after adjusting for sociodemographic characteristics, smoking history, and mental health status, underlining the substantial impact of COPD on perceived health and quality of life. By demonstrating that brief, single-item wellbeing measures consistently differentiate those with COPD from the general population, our findings support their construct validity as indicators of chronic disease burden in population health surveillance. As public health systems increasingly prioritise person-centred outcomes and seek efficient ways to monitor health inequalities, these measures offer a valuable and scalable tool for capturing the lived experience of illness. Further research is warranted to assess their responsiveness to intervention and applicability across other long-term conditions.

## Data Availability

The data analysed in this study are publicly available. https://ukdataservice.ac.uk/find-data/browse/health/.
